# AI-assisted IVF company websites: a content analysis of marketing claims, information quality, legal and ethical issues

**DOI:** 10.1093/hropen/hoag086

**Published:** 2026-09-30

**Authors:** Li Du, Yinuo Zhang

**Affiliations:** Faculty of Law, University of Macau, Taipa, Macau SAR, China; Asia-Pacific Academy of Economics and Management, University of Macau, Taipa, Macau SAR, China; Faculty of Law, University of Macau, Taipa, Macau SAR, China

**Keywords:** assisted reproduction, artificial intelligence, direct-to-consumer advertising, transparency, health decision-making, privacy, data rights, benefits and risks, patient education, health and AI literacy

## Abstract

**STUDY QUESTION:**

How do providers of AI-assisted IVF products and services present their AI systems, characterize their products and services, and address patient privacy and data protection concerns on their websites?

**SUMMARY ANSWER:**

Websites of AI-assisted IVF providers predominantly emphasize benefits while failing to disclose risks, provide insufficient information about AI systems and data sources, and inadequately address privacy protection, with none of them including AI-specific provisions in their privacy policies.

**WHAT IS KNOWN ALREADY:**

Websites promoting AI-assisted products and services for IVF treatments are a key information source for patients, can shape perceptions about this advanced technology, and have an impact on patients’ treatment decisions. However, the way providers present such services on their websites has not been previously studied.

**STUDY DESIGN, SIZE, DURATION:**

Using online searches and a review of research abstracts presented at the 2022 ESHRE annual meeting, we identified 21 companies offering AI-assisted IVF products or services. Website content was captured between 15 and 22 August 2023, and content analysis was done between 25 August and 15 September 2023. In response to an external reviewer’s recommendation, we did a follow-up review on 30 June 2026.

**PARTICIPANTS/MATERIALS, SETTING, METHODS:**

Twenty-one companies (7 IVF equipment producers, 14 exclusively developing AI-assisted IVF products) based in Europe (n = 9), Israel (n = 4), Canada/USA (n = 5), and China (n = 3) were evaluated in this study. The companies collectively offered 30 AI tools including embryo quality assessment, oocyte quality assessment, sperm analysis, endometrial receptivity analysis, and ovarian stimulation optimization. A subset of 10 companies’ websites was used for thematic content analysis and the resulting themes were used to develop a coding book with 22 questions examining (i) benefit and risk descriptions; (ii) AI system explanations and performance; (iii) data protection and privacy issues; and (iv) portrayal of products/services. The coding book was finalized and subsequently applied by one of the authors to complete an analysis of the entire dataset. Based on an external reviewer’s suggestion, we also analyzed the AI models on providers’ websites. Independent coding of 60% of samples achieved almost perfect inter-coder agreement (Cohen’s Kappa: 0.831–1.000).

**MAIN RESULTS AND THE ROLE OF CHANCE:**

All 21 websites (100%) mentioned benefits of AI-assisted IVF, with the most frequently cited benefit being ‘increased accuracy’ (specific to each provider’s product or service) (61.9%, n = 13). However, no websites explicitly outlined potential risks associated with AI use. Twenty companies (95.2%) described their AI systems, yet only 33.3% (n = 7) disclosed sample sizes used for AI development, and only 23.8% (n = 5) indicated dataset diversity or representativeness. Although 13 websites (61.9%) provided links to privacy policies, none included provisions specifically addressing patient health data processing when using AI products or services. Eight websites (38.1%) mentioned data protection measures on product introduction pages (e.g. encryption, de-identification), with five referencing GDPR compliance and three referencing HIPAA compliance. Nine companies (42.9%) marketed products as unique or ‘first’ in the IVF industry; six (28.6%) mentioned patent information. Nineteen companies (90.5%) required clinician oversight or final decision-making, while two companies employed direct-to-consumer (DTC) marketing models.

**LIMITATIONS, REASONS FOR CAUTION:**

The analysis was limited to 21 companies identified through the ESHRE 2022 annual meeting and Google searches and may not fully reflect all concerns associated with AI-assisted IVF products. Findings reflect documented website claims rather than independently verified compliance status. The analysis did not capture the technical development, supply chain, or hosting arrangements of the AI tools. Website content is dynamic and may have changed since data collection.

**WIDER IMPLICATIONS OF THE FINDINGS:**

The study reveals limited transparency of AI systems, unsubstantiated claims about AI efficacy for improving IVF success rates, and downplaying of relevant risks on provider websites. This inaccurate portrayal may distort patient expectations and compromise informed decision-making. The DTC AI-assisted IVF products—a model in which AI product suppliers bypass professional healthcare providers to market and sell directly to consumers—carry heightened risks for patients. The study advocates that AI providers (i) adhere strictly to truthful advertising regulations with balanced risk disclosure; (ii) proactively disclose AI system information through layperson-friendly explanations and separate AI-specific privacy policies; and (iii) establish ethics review mechanisms to verify claims and develop patient-centric communication materials. These measures are essential for responsible AI communication in reproductive medicine.

**FUNDING:**

This study was supported by the University of Macau via the MYRG project MYRG2023-00025-FLL and APAEM/SG/0002/2025.

**DISCLOSURES:**

Both authors declare that there are no conflicts of interest.

**TRIAL REGISTRATION NUMBER:**

N/A

WHAT DOES THIS MEAN FOR PATIENTS? Websites promoting artificial intelligence (AI)-assisted products and services for IVF treatments are a key information source for patients, can shape perceptions about this advanced technology, and have an impact on patients’ treatment decisions. However, the way providers present such services on their websites has not been previously studied.

This study examines the overall portrayal of AI-assisted IVF and key issues that may influence patients’ understanding and treatment decisions. Specifically, it investigates how providers explain their AI systems, how they characterize their products and services, and how they address patient privacy and data protection concerns.

Using online research and supplementary data from the European Society of Human Reproduction and Embryology (ESHRE), we identified 21 companies offering AI-assisted IVF products or services. We then conducted a thematic content analysis of the publicly available information on their websites between 15 and 22 August 2023. We also conducted a follow-up review on 30 June 2026.

Our study indicates that while the websites emphasized the benefits of AI-assisted IVF, they did not discuss risks associated with the use of AI in this context. We established that 33.3% of the websites provided information about the source and quality of data used by AI. Although 38.1% of the websites addressed data protection matters, the privacy policies of all 21 providers were silent on the use of AI.

The results of our analysis have shown limited transparency of AI systems, false claims about AI tool efficiency for IVF success rates, and descriptions that downplay relevant risks. This inaccurate portrayal could distort expectations and present challenges to both patients and healthcare providers. The direct-to-consumer (DTC) AI-assisted IVF products—a model in which AI product suppliers bypass professional healthcare providers to market and sell directly to consumers—carry heightened risks for patients. This study advocates for a proactive involvement of AI providers to educate prospective patients and the public and adhere to advertising regulations. Additionally, we recommend establishing ethics review mechanisms, having a third-party ethics board verify claims before they are published and develop patient-centered communication materials.

## Introduction

For patients struggling with infertility, ART offers significant hopes and possibilities for having genetic offsprings. In conventional procedures, patients need to undergo evaluations at multiple stages during the IVF cycle. For example, before cycle initiation, a standard fertility evaluation is performed through blood tests, ultrasound examinations, and semen analysis to assess a patient’s fertility potential. Following this pre-cycle assessment, women undergo controlled ovarian stimulation to promote the development of multiple follicles. Egg retrieval is then performed, and, in cases of male factor infertility, sperm selection may be used. Finally, embryo quality is evaluated to select the most viable embryo(s) for transfer, thereby improving the chances of a successful pregnanc*y* (Mancuso *et al.*, 2026). However, these evaluations are influenced by the expertise and subjective assessments of specialists, and hence evaluation quality remains inconsistent (Balaban and Gardner, 2019). Given these potential limitations of current assessment methods, AI technology is being increasingly integrated into the IVF procedure (Hanassab *et al.*, 2024).

A growing number of studies have demonstrated the potential value of using AI in improving IVF process efficiency. AI may improve ovarian stimulation protocols tailored for specific patients, enable gamete and embryo selection, reduce evaluation time, and increase overall efficiency of IVF procedures (Buldo-Licciardi *et al.*, 2023; Goss *et al.*, 2024; Wang *et al.*, 2024; Wygocki *et al.*, 2024; Cimadomo *et al.*, 2025; Hanassab *et al.*, 2025; Olawade *et al.*, 2025). Specifically, predictive machine learning systems based on patient personal health data, such as age, antral follicle count, and hormone levels can potentially improve the accuracy of ovarian stimulation protocols and provide personalized stimulation dosing, which is critical to IVF success. Functional biomarkers of fertility combined with optical imaging and deep learning may improve sperm selection and embryo evaluation (Chow *et al.*, 2021). Preimplantation genetic testing, including polygenic embryo screening, has also been integrated into AI-enabled embryo selection (Widen *et al.*, 2022; Raz *et al.*, 2026). By using big data analytics and polygenic risk scores (PRS), polygenic embryo screening offers potential benefits in rapidly identifying embryos with the best health prospects (Capalbo *et al.*, 2024). Generative AI is also being actively explored, both to predict stimulation protocols and to develop personalized prediction models for clinical pregnancy (Cao *et al.*, 2024, 2025). However, generative AI has not yet been considered sufficiently reliable for clinical use (Dinç *et al.*, 2026).

In clinical practice, several AI tools have received regulatory clearance and entered the European market. However, studies have established no evidence for the clinical efficiency of such AI tools, and comprehensive and large-scale clinical validation must precede any routine integration of AI into IVF practices (Correa *et al.*, 2024; Illingworth *et al.*, 2024; Olawade *et al.*, 2025). The Human Fertilisation and Embryology Authority (HFEA) has stated that AI-based add-ons do not generate better clinical outcomes. Based on current evidence, claims of AI superiority are premature and potentially misleading.

Beyond the limited evidence supporting AI-assisted IVF tools over conventional ones, scholars have also raised ethical and legal concerns associated with the use of AI in IVF. For example, some studies have argued that the predictive machine learning algorithms carry risks related to data bias, leading to different performance across diverse patient populations (Koplin *et al.*, 2025). Deep learning tools for evaluating embryos commonly operate as ‘black boxes’, posing challenges for physicians in explaining decision-making rationales and communicating the limitations and risks of treatment options (Koplin *et al.*, 2025). In this context, patients’ autonomy and right to informed consent may not be fully safeguarded (Anastasi *et al.*, 2023; Koplin *et al.*, 2025). The use of generative AI in IVF poses additional risks, including hallucinations, fabrications, and incorrect or misleading statements, which directly undermine the accuracy of the information and generate incorrect recommendations, thereby potentially harming patients’ right to health.

Moreover, under the EU *Artificial Intelligence Act* (AIA), AI systems that aid decision-making in IVF treatments, such as ovarian stimulation and embryo implantation, by providing decision-relevant information, would in principle be classified as high-risk. Providers of such systems must conform to stringent legal requirements, including enhanced transparency obligations, mandatory medical device conformity assessment, and the establishment of quality management systems. These measures aim to ensure the trustworthiness and safety of the AI systems while respecting patients’ fundamental rights.

Alongside these binding regulatory frameworks, broader ethical guidance has also emerged from international bodies. The World Health Organization (WHO), the United Nations Educational, Scientific and Cultural Organization (UNESCO), and other organizations have issued advisory guidelines on the ethical principles of AI (UNESCO, 2021). These guidelines complement domestic AI laws by calling on AI developers, providers, and healthcare service providers to take appropriate measures to protect human autonomy, ensure transparency and explainability, uphold accountability, and promote fairness in AI systems (WHO, 2021).

In addition, some AI products and services that support IVF treatments are offered directly to consumers without the involvement of physicians. Examples of direct-to-consumer (DTC) AI-assisted IVF products and services include: at-home fertility tests linked to an AI-driven semen testing microscope, personalized prediction reports on the chances of conceiving a baby via IVF, and mobile applications offering insights to both clinics and patients at each point of care in an assisted reproduction cycle. Research exploring DTC marketing methods across biomedical industries has established that such marketing practices, particularly when they exaggerate benefits or downplay risks, can inadvertently erode public trust in proven, safe, and effective alternative therapies that compete with the advertised DTC products (He *et al.*, 2023). For example, in the pharmaceutical field, it has undermined patients’ trust in physicians’ recommendations and has often led them to ignore professional advice and guidelines (Wang and Kesselheim, 2013; Perrotta and Hamper, 2023; Copp *et al.*, 2024; Nagappan *et al.*, 2024; Rodeo, 2024; Makary, 2025).

In the digital age, websites of AI service providers function as a critical information bridge between providers and the public (Bergmo *et al.*, 2023; Mayette *et al.*, 2024). This remains true in AI-assisted IVF. While clinicians may disclose AI-related information during the informed consent process, the information on websites shapes prospective patients’ initial perceptions of AI applications in IVF (Brochu *et al.*, 2019). Many individuals seeking IVF treatment conduct online searches on assisted reproductive technologies and services before their initial clinic visit (Perrotta *et al.*, 2025). Furthermore, even though patients regard physicians as their most trusted source, they actively turn to online sources when their informational needs remain unmet by clinicians (Mayette *et al.*, 2024). In addition, in cases where a clinic does not offer AI-assisted IVF services, patients cannot learn about such services during consultations (Shoham *et al.*, 2025), and provider websites may arguably serve as a primary gateway for patient engagement with these technologies. As previous research indicates, website information can directly or indirectly influence patients’ perceptions of AI tools and their decisions regarding innovative treatments (Alkazemi and Van Stee, 2020; Lensen *et al.*, 2021; Copp *et al.*, 2024; Gojanur *et al.*, 2024).

Given this influence, it is worth examining how providers of AI-assisted IVF describe and market such products and services on their websites. Specifically, it is important to look at how they explain their AI systems, whether they clarify data collection procedures and sources of the AI products, and whether they address privacy and data protection issues relevant to patients (Shevtsova *et al.*, 2024). This study aims to answer these questions by undertaking a content analysis of providers’ websites to elucidate the current state of AI-related information disclosure and marketing practices directed at the public and prospective patients. Legal and ethical concerns surrounding AI-assisted IVF encompass risks to patient autonomy, informed consent, reproductive interests, and data privacy. Our analysis focused specifically on whether online marketing practices involve such risks, the novel challenges they present, and the pathways to ensure responsible, patient-facing communication.

## Materials and methods

From 15 August to 22 August 2023, we used google.com and keywords such as ‘artificial intelligence’ and ‘in vitro fertilization’, ‘AI’ and ‘IVF’, ‘AI’ and ‘assisted reproductive technology’, ‘AI’ and ‘embryo’, ‘AI’ and ‘sperm’, ‘AI’ and ‘endometrial receptivity’, and ‘AI’ and ‘oocytes’ in both English and Chinese to search for providers that offer AI-assisted IVF products and services. Based on this online research, we identified 20 companies. We then excluded one company from our dataset, leaving 19 companies, because its product reportedly using AI for sperm counting was not featured or described on the company’s website. Besides the Google search, we also reviewed research abstracts and posters from the 38th Hybrid Annual Meeting of ESHRE (ESHRE, 2022), which were provided on ESHRE’s website. This review led us to identify four other companies using AI tools in IVF. For these four companies, we found that AI systems developed by two companies were still in the research phase. Consequently, we removed these two companies from our dataset. Therefore, a total of 21 companies were included in our dataset (see Table 1). These websites marketed products to both Healthcare Professionals (HCPs) and consumers. Company names and product names have been coded throughout this study. All references to company and product names appearing in the original website content quoted in the tables have been replaced with their corresponding codes to ensure consistency and anonymity.

**Table 1.** Overview of companies and AI tools deployed.

Company identifier	Product name	Stated AI type	Product function	Clinician oversight or decision required	Locations/headquarters	Investigation time
IVF-P1	P1-1	Deep learning	Embryo quality assessment and selection	Require clinician final decision-making	Sweden	22 August 2023
IVF-P2	P2-1	Computer vision	Embryo quality assessment and selection	Require clinician final decision-making	Israel	22 August 2023
	P2-2	Deep learning Computer vision	Oocyte quality assessment	Require clinician final decision-making		
IVF-P3	P3-1	Unspecified ‘AI’	Embryo quality assessment and selection	Require clinician final decision-making	USA	22 August 2023
	P3-2	Deep learning	Embryo quality assessment and selection	Require clinician final decision-making		22 August 2023
	P3-3	Deep learning	Embryo quality assessment and selection	Require clinician final decision-making		
	P3-4	Deep learning	Oocyte quality assessment	Require clinician final decision-making		
	P3-5	Unspecified ‘AI’	Endometrial receptivity analysis	Require clinician final decision-making		
IVF-P4	P4-1	Machine learning (scope undefined)	Embryo quality assessment and selection	Require clinician final decision-making	Israel	22 August 2023
IVF-P5	P5-1	‘Multiple neural network architectures’	Embryo quality assessment and selection	Require clinician final decision-making	China	15 August 2023
IVF-P6	P6-1	Deep learning	Embryo quality assessment and selection	Require clinician final decision-making	Israel	22 August 2023
IVF-P7	P7-1	Deep learning	Embryo quality assessment and selection	Require clinician final decision-making	UK	22 August 2023
	P7-2	Computer vision	Sperm-selector assistant	Require clinician final decision-making		
IVF-P8	P8-1	Unspecified ‘AI’	Embryo quality assessment and selection	Require clinician final decision-making	Spain	22 August 2023
IVF-P9	P9-1	‘Mathematical algorithms and machine learning technology’	Embryo quality assessment and selection	Require clinician final decision-making	Denmark	16 August 2023
IVF-P10	P10-1	Unspecified ‘AI’	Embryo quality assessment and selection	Require clinician final decision-making	China	17 August 2023
IVF-P11^*^	P11-1	Unspecified ‘AI’	Embryo quality assessment and selection	Require clinician final decision-making	France	22 August 2023
IVF-P12	P12-1	Unspecified ‘AI’	Embryo quality assessment and selection	Require clinician final decision-making	German	22 August 2023
IVF-P13	P13-1	‘Machine learning’ (scope undefined) Computer vision	Endometrial receptivity analysis	Require clinician final decision-making	Canada	17 August 2023
IVF-P14	P14-1	Machine learning (scope undefined)	Endometrial receptivity analysis	Require clinician final decision-making	China	22 August 2023
IVF-P15	P15-1	Unspecified ‘AI’	Oocyte quality assessment	Require clinician final decision-making	Canada	22 August 2023
	P15-2	Unspecified ‘AI’	Oocyte quality assessment	Require clinician final decision-making		
IVF-P16	P16-1	Unspecified ‘AI’	Assisted ovarian stimulation	Require clinician final decision-making	Poland	17 August 2023
	P16-2	Unspecified ‘AI’	Embryo quality assessment and selection	Require clinician final decision-making		
IVF-P17	P17-1	Unspecified ‘AI’	Oocyte quality assessment	Require clinician final decision-making	Portugal	22 August 2023
IVF-P18	P18-1	Unspecified ‘AI’	Assisted ovarian stimulation	Require clinician final decision-making	USA	22 August 2023
	P18-2	Unspecified ‘AI’	Embryo quality assessment and selection	Require clinician final decision-making		
IVF-P19^*^	P19-1	Unspecified ‘AI’	Sperm analysis (DTC AI-assisted IVF products)	No oversight	France	22 August 2023
IVF-P20	P20-1	Unspecified ‘AI’	Personalized fertility treatment APP for patients (DTC AI-assisted IVF products)	No oversight	Israel	22 August 2023
IVF-P21	P21-1	Machine learning (scope undefined)	Provide reports about the probability of having a baby with IVF (DTC advertising)	Require clinical oversight	USA	22 August 2023

* Due to the lengthy process of peer review, we looked the updated versions of the website on June 30, 2026, and these two websites are currently not accessible.

We captured descriptions and marketing information of AI-assisted IVF products and services from providers’ websites and downloaded privacy terms from 13 companies. Between 25 August and 10 September 2023, one author conducted a preliminary, inductive review of website content and privacy terms from a subset of 10 websites to develop the analytical framework. This exploratory review aimed to identify recurring themes, patterns, and issues emerging directly from the data. Based on these inductive observations, we developed a coding book consisting of 22 questions examining: (i) whether websites described the benefits and risks of AI; (ii) whether websites explained the AI systems and their performance; (iii) whether data protection and privacy issues were mentioned; and (iv) how websites portray their AI products and services. The coding book was finalized and applied by one author to the entire dataset without further modification. Questions 3 through 22 employed a binary classification approach, while the first two questions explored the benefits and risks mentioned on the websites. In response to an external reviewer’s recommendation, we additionally analyzed the AI models described on providers’ websites on 30 June 2026.

To minimize potential interpretation bias and evaluate coding reliability, an independent coder with expertise in international law used the same coding book to analyze approximately 60% of randomly selected samples from our dataset. The website content, service terms, privacy policies, and related content were reviewed. Subsequently, we utilized IBM SPSS Statistics (IBM Corp., Armonk, NY, USA) to calculate Cohen’s Kappa (K) to compare the consistency of the two rounds of coding. The coding results from both coders were input into the software, and then the Kappa statistic was computed for each question to assess the level of agreement between the two coders. The results for all coding questions ranged from 0.831 to 1.000. According to the benchmarks established by Landis & Koch, a Kappa value greater than 0.80 indicates almost perfect agreement (Landis and Koch, 1977). This indicates that we obtained almost perfect agreement scores.

## Results

### Basic information about AI in the IVF practice

Among the 21 companies, seven are IVF equipment producers, and 14 are exclusively developing AI-assisted IVF products. Regarding the companies’ locations, nine are based or headquartered in countries in Europe, four in Israel, five in Canada and the USA, and three in China. With respect to their target clients, 18 companies are oriented towards healthcare providers and organizations, offering products intended for professional use. The remaining three companies employ the DTC marketing model, with two of them promoting products or services that are designed for patients’ use during the IVF treatment process. Excluding two fully DTC AI-assisted IVF products or services, the use of the remaining 28 AI tools (93%) requires either clinical physician supervision or final decision-making by physicians based on AI-generated results.

### Explanations of AI systems

Twenty companies (95.2%) described their AI systems. For example, the AI systems for embryo evaluation and selection were typically described as utilizing deep learning tools for the analysis of embryonic development images, and the resulting analysis was presented as a range of scores that could indicate the likelihood of a successful implantation. A clinician would make the final decision for embryo transfer based on a score generated by AI.

Aside from textual explanations, companies have used other methods to explain their AI systems, e.g. displaying images of the AI software’s interface on the website (n = 17, 81.0%) or using short videos to introduce the product’s functions (n = 11, 52.4%). Fifteen websites (71.4%) stated that their AI products had been validated through published studies, or they had conducted and published retrospective studies on the clinical validation of their AI-assisted IVF products. Additionally, 13 websites (61.9%) created dedicated pages to feature relevant scientific research on their products or to present online lecture videos related to AI-assisted IVF products.

All 21 websites provide contact channels. Among these, five providers include a ‘patient’ option in their contact forms for clients who want to acquire further information about their products and services. Additionally, three companies have offered downloadable manuals detailing product usage.

Seven providers (33.3%) revealed the sample size they utilized for developing their AI products. Five websites (23.8%) indicated the diversity, representativeness, and comprehensiveness of datasets employed for AI training, validation, and testing. Moreover, five other providers (23.8%) explained transparency and interpretability of the algorithms they developed. For instance, one company claimed that the biological parameters that contribute to final outcomes are explained to clinicians and patients in a comprehensible and transparent manner, solving the ‘black box’ issues of AI (see Table 2).

**Table 2.** Examples of descriptions of AI systems on the websites.

The content mentioned by the websites	Total number	Examples
Textual description of AI system	20	‘The application automatically identifies high-quality embryos based on a single image. The algorithm learned to recognize the best embryos from an immense library of human embryo images.’—IVF-P16‘During IVF, doctors make many decisions that will affect a cycle’s outcomes including medication doses and timing for procedures. IVF-P18’s AI software helps doctors review what has worked for patients similar to you. With this information, your doctor can make the best decisions for your care.’—IVF-P18
Description of sample size in AI development	7	‘Our software has analyzed over 100,000 egg images and their outcomes to learn patterns that help predict if your egg will become a healthy embryo (blastocyst).’—IVF-P15
Description of the diversity and completeness of the dataset	5	‘Algorithms trained on large, diverse datasets collected from IVF clinics all around the world to prevent bias’ and ‘Clinical research has been performed spanning across a broad spectrum of institutions worldwide varying in size and population diversity.’—IVF-P2
Description of algorithmic explainability or transparency	5	‘Our solutions provide fully interpretable results. You and your patients surely will understand them.’—IVF-P16 ‘We have opened the proverbial “black box” of AI to explain how AI models get to a decision, so you can implement XXX safely.’—IVF-P2

### Benefits and risks of using AI in IVF treatment

While all companies mentioned the benefits of using AI in IVF treatments, none explicitly outlined potential risks associated with its use. We have identified 10 benefits as stated by the companies (see Table 3). The most frequently cited benefit (n = 13, 61.9%) pertains to ‘increased accuracy’. The specific components of ‘accuracy’ are determined by the functionality of the product and can be categorized as follows: (i) accuracy in prediction, such as accurately predicting embryo development, implantation potential, and human chorionic gonadotropin (beta-hCG) results; and (ii) accuracy in measurement, which specifically pertains to precise measurements of follicles and sperm.

**Table 3.** Benefits of using AI in IVF treatments.

Benefits		Total number		Examples
**Benefits**				
Accuracy	Accuracy in prediction or evaluation	7	13	‘Blastocyst prediction accuracy of 96% and 73% prediction accuracy for implantation prediction’—IVF-P2‘P11-1 also generates a personalized embryo transfer recommendation for increased accuracy of the chances an embryo has to lead to a clinical pregnancy.’—IVF-P11‘Our AI performs at 97% accuracy with less than a 20% error margin—validated against WHO standards & ISO 15189-compliant labs such as the Karolinska Institute in Sweden.’—IVF-P19‘P9-1 is the only test on the market using artificial intelligence (AI) based on clinical outcome data of live births and sustained pregnancy outcomes, eliminating subjectivity, and improving accuracy.’—IVF-P9
	Accuracy in measurement	2		
	Accuracy without specifying content	4		
Improve workflow standardization and efficiency		10		‘Streamline laboratory workflow‘—IVF-P13‘One platform to streamline the entire IVF process from end to end.’—IVF-P18
Shorten time to pregnancy and save costs		6		‘Minimize time to pregnancy with our AI-based score.’—IVF-P11
Non-invasive		6		‘Non-invasive, no hardware—The AI uses standard microscope images of embryos.’—IVF-P3
Patient-friendly		6		‘Cut through the jargon with an easy-to-understand report built for you that equips you and your doctor to make the best decisions‘—IVF-P15
Boost the success rate of IVF		5		‘Clinically tested embryo selection software aimed to increase IVF success rates by an average of 15%.’—IVF-P4‘Over 350,000 embryos have been tested with our robust P9-1 platform shown to improve the odds of pregnancy and IVF success.’—IVF-P9
Objective and consistent evaluation/prediction		5		‘Consistent approach to embryo evaluation. Day to day differences in staffing, or introduction of new embryologists will not affect the way embryos are scored.’—IVF-P1
Easy to use		5		‘Now you can get an objective ranking based on the likelihood of achieving a fetal heartbeat on days 2, 3 or blastocyst stage of each of a patient’s embryos at once—with just a touch of a button.’—IVF-P1‘All at once, at the touch of a button.’—IVF-P4
Avoid human error and unconscious bias		4		‘Insights that are 100% repeatable—no risk of human error, subjectivity, or variability’—IVF-P15
AI outperforms experts in making predictions		4		‘P16-1 detects 93% of large follicles compared to highly skilled professionals.’—IVF-P16‘38% more accurate than embryologist alone.’—IVF-P6
**Other benefits**		♦ ‘improve IVF/ART outcomes’♦ ‘promote the birth rates’♦ ‘increase the probability of pregnancy’ or ‘increase pregnancy rates’♦ ‘makes IVF success and costs more predictable’		

Ten providers (47.6%) underlined the advantage of improving the standardization and efficiency of the workflow in laboratories and clinics. Six providers stated that the use of AI can enable patients with a shortened time-to-pregnancy and potential cost savings. Four companies mentioned that their AI offerings can help avoid human error and unconscious bias, while another four claimed that AI outperforms experts in making predictions.

### Privacy and data protection issues

Although 13 providers provided a link to their privacy policies, these privacy policies mainly covered provisions related to website access and operation. None of the websites provided privacy policies that included provisions related to the processing and protection of patients’ health data when using AI products or services.

Eight websites (38.1%) mentioned the measures they had taken for data protection when using AI-assisted IVF products directly on their websites’ user interfaces (rather than in privacy policy sections), such as encrypting and securely hosting patient data in local areas, using anonymized or de-identified methods, or adopting decentralized machine learning solutions. Five of these websites mentioned that their AI solutions met the requirements of the General Data Protection Regulation (GDPR), and three mentioned that they met the requirements of the Health Insurance Portability and Accountability Act (HIPAA).

Three companies (14.3%) distinctly addressed the issues of privacy and data protection during the application of AI in IVF-related scientific research. They detailed matters such as the objectives and types of data collected, the methods employed to protect privacy during data processing, and the legal grounds for data processing (see Table 4). Out of these three companies, two addressed these concerns in their privacy policies, while one elaborated on these issues on its website.

**Table 4.** Descriptions of privacy and data protection issues on the websites.

The content mentioned by the websites	Total number	Examples
Websites that mentioned the issues of privacy and data protection during the application of AI in IVF-related scientific research	3	‘IVF-P11, as the promoter of the study, is “responsible for the processing” and guarantees that this processing is carried out in complete safety and in accordance with the local regulations in force, in particular the “Informatique et Libertés” law of 6 January 1978 as amended and to European Regulation No. 2016/679/EU of April 27, 2016 (the GDPR). To ensure this, IVF-P11 has appointed a personal data protection officer.’—IVF-P11‘We obtain de-identified clinical data from research partner IVF clinics (“Research Partner Data”). This dataset includes embryo images and videos, as well as clinical datapoints, such as BMI, age, drug treatment protocols, reason of infertility, PGT and IVF treatment results. Research Partner Data does not include direct identifiers such as patients’ names or contact information. This data is used by us only for scientific research and training of our algorithms, and is not used in order to produce automated decision making regarding the data subjects. Note, clinical data we process as Processors on behalf of our clients as part of our Services does not constitute part Research Partner Data and is not the subject of this policy.’—IVF-P2‘Purpose: We use the above data in an anonymous, aggregated or pseudonymized manner to assess your suitability for clinical research and to invite you to partake in clinical research with one of our clinical research partners. For the avoidance of doubt, we do not pass on any personal data to our clinical research partners without your explicit consent.’—IVF-P20
Websites on product introduction web pages mentioned information about privacy and data protection concerning the use of AI products or AI services	8	‘IVF-P3 is both HIPAA and ISO13485 compliant. Local cloud servers are used in each region such that your data never leaves the region of origin, meeting strict data privacy requirements including GDPR. Data is always encrypted in the application to the highest standards.’—IVF-P3

### Promotion of services

Nine companies (42.9%) marketed their products as unique or claimed that they were the first in a particular area of the IVF industry. For example, there were claims about ‘the first and only transparent AI-based decision support system’. For example, one company claimed it was ‘the only non-invasive diagnostic test available to ART clinicians to assess endometrial receptivity’. Three companies stated that their AI software was expected to be or could be applied to ‘all clinics’ or ‘all patients’.

Six companies (28.6%) mentioned patent information about their products to enhance marketing credibility. Among them, only one website explicitly stated that the type of patent was an innovation patent, while the other five companies did not specify the exact type of patent (see Table 5).

**Table 5.** Promotion of services and products.

Promotion of services and products	Total number	Company		Examples
Statements about the first developer in a specific field of this industry or about that the product is unique	9	Mentioned directly in the product promotional interface of the websites	IVF-P2	‘the first and only transparent AI-based decision support system that provides IVF professionals with visibility into the clinical and laboratory parameters that make up data output’
			IVF-P20	‘the first ever App specifically designed to empower and support women going through fertility treatments’
			IVF-P9	‘the only test on the market using AI based on clinical outcome data of live births and sustained pregnancy outcomes, eliminating subjectivity, and improving accuracy’
			IVF-P13	‘the only non-invasive diagnostic test available to ART clinicians to assess endometrial receptivity’
			IVF-P19	‘UK’s first virtual male fertility clinic’
			IVF-P21	‘IVF-21 is the only highly-scalable AI platform to provide scientifically-validated, personalized reports that counsel patients from diverse demographics about their probability of having a baby with IVF.’
		Mentioned such information in the news and posters displayed on the websites	IVF-P3	‘IVF-P3 commended by Frost & Sullivan for its first-of-a-kind cloud-based IVF image analysis software solution’
			IVF-P6	‘the first fully operational AI -based IVF software platform’
			IVF-P15	‘trail blazing-the first and only way to measure oocyte quality-fertility treatment’s missing piece’
Websites that mentioned that the product can be generally applied to all clinics/patients	3	IVF-P1		Automatic embryo scoring by AI shows convincing results for all provided subgroups of data and can be expected to generalize well to all clinics.
		IVF-P3		‘Accessible for clinics of any size, anywhere’
		IVF-P17		‘Recomendado para todas as pacientes’
Websites that mention patent information	6	IVF-P7		‘2019 Patent is submitted for P7-1. 2020 Preliminary patent for an AI assisted sperm selection for ICSI is submitted. 2021 P7-2 gets CE Mark, and a definitive Patent is submitted.’
		IVF-P12		‘The innovative, patented P12-1 software is now available in our award-winning benchtop incubator XXX.’
		IVF-P14		‘The endometrial tolerance test P14-1 ‘A method for determining endometrial tolerance and its application’ was granted a national patent.’
		IVF-P10		This website shows pictures of original technology international patents about the P10-1
		IVF-P21		‘US Patent Number 9,458,495B2, Foreign Counterparts and Patents Issued’
		IVF-P15		‘P15-1: US10,552,957; US10,748,288. Other patents pending. P15-2: US10,552,957; US10,748,288. Other patents pending.’

## Discussion

Our study has identified several significant legal and ethical issues on websites that market AI-assisted IVF products and services. These issues pertain to inadequate AI system explanation, portrayal of AI-assisted tools, and privacy protection. Specific concerns include: flawed explanation of AI-assisted IVF systems, violations of marketing compliance, and failure to disclose inherent risks and limitations of AI applications. These issues may pose risks to patient autonomy, constitute false advertising, and mislead patients’ understanding of the technology. We therefore propose a mechanism for responsible AI communication to bridge the identified gaps.

### Undermining patient autonomy

Protecting patient autonomy in AI-assisted IVF treatment is crucial. It ensures individuals retain meaningful control over their reproductive decisions (Armstrong *et al.*, 2023; Pugeda, 2024). However, this control can be undermined by the design and mode of operation of AI systems. For example, suppose an AI model identifies non-medical genetic traits as predictors of implantation success and systematically prioritizes embryos possessing those features. If this selection criterion is not disclosed, the AI’s recommendation could override a patient’s preference against selecting embryos based on non-medical traits, leading to outcomes that contradict their choices (Afnan *et al.*, 2021b; Liu *et al.*, 2022).

The foundation for autonomous, rational reproductive health decision-making lies precisely in ensuring access to accurate information (Rubinelli, 2025). However, our study has indicated that, although 95% of companies described the functions and application scenarios of AI, providers did not offer sufficient information about the AI systems. For example, providers that only mentioned that they use ‘embryo images’ and ‘oocyte images’ to train the AI models did not disclose information about the sources and quality of data for AI algorithm training, nor did they explain specific factors that impact AI decision-making results. Without such information, it would be difficult for users to assess potential adverse consequences of biased predictions (Ferrara, 2024).

Our findings are aligned with previous empirical studies on AI in healthcare. For example, Daus *et al.* found that patients are frequently neither informed nor asked to consent to AI use by physicians (Robbins and Brodwin, 2020; Daus *et al.*, 2026). Yet, transparency and explainability of AI can safeguard patient autonomy to make informed decisions regarding AI-assisted IVF services. These principles help patients question, override, or opt out of AI-driven recommendations. In fact, AI tools’ transparency and explainability have been acknowledged as fundamental principles by numerous ethics and regulation initiatives within the domain of AI governance. For example, WHO has identified transparency, together with explainability and intelligibility, as a core principle for the use of AI in healthcare (WHO, 2021). The EU AIA also prioritizes the principle of transparency for AI technology providers to guarantee accountability and informed decision-making.

In clinical settings, physicians have a duty to inform patients about AI usage, including specific applications and potential risks, in order to meet the requirements for meaningful informed consent in IVF care. However, physicians’ explanatory capacity could be inherently limited by technical knowledge gaps and the black box nature of algorithms (Tamir, 2023). This technical constraint does not diminish but rather underscores the critical role of AI providers in fulfilling their transparency and explainability obligations to IVF professionals and patients by supplying accurate information about their AI-assisted IVF systems. For example, by revealing biologically meaningful parameters used in embryo assessment, physicians can better articulate treatment rationales and better inform patients during the consent process. Moreover, disclosing the factors and logic behind AI-generated results can help patients understand the reasoning behind recommendations regarding embryo selection or success predictions, thus strengthening their capacity for informed choices and safeguarding their autonomy in reproductive decisions (Afnan *et al.*, 2021a; Tamir, 2023).

However, our study indicates that the majority of websites did not provide clear explanations of the AI systems used in IVF procedures. Although some websites cited scientific research and publications to explain AI-assisted products, this kind of highly specialized technical information is likely of little help to laypersons trying to understand how complex AI systems operate. In addition, most consulting services on the websites are available only to healthcare professionals. For companies that provide DTC AI products, consulting channels exist but are not specifically designed to explain AI systems.

Given that explanations about AI systems on provider websites may influence patients seeking IVF treatments, the disclosures and explanations by service providers are critically important. Therefore, we recommend that providers of AI-assisted IVF services communicate technical information to clinicians and patients in a clear and accessible manner. For instance, they could develop short, animated videos or visual process charts to explain how AI technology is incorporated into the IVF treatment cycle and the protocols for collecting, using, and storing patient data. These visual communication tools would enable patients to make better-informed decisions, thereby strengthening their autonomy in IVF decision-making (Afnan *et al.*, 2021a; Tamir, 2023).

### False advertising issues

There has been no established evidence supporting AI efficacy in IVF. However, our website analysis revealed specific promotional claims that directly contradict this evidence base. Specifically, our study indicates that the most frequently cited benefit by providers (n = 13, 61.9%) is the increased accuracy of AI, with claims such as ‘96% prediction accuracy’ or increasing ‘IVF success rates by an average of 15%’. Critically, this is not merely an exaggeration of benefits. Previous studies have shown that randomized controlled trials (RCTs) of AI application in IVF, specifically embryo selection (the use of AI-based computer vision) and ovarian stimulation personalization (the use of machine learning prediction), have not generated meaningful improvement in actual clinical outcomes (Correa *et al.*, 2024; Illingworth *et al.*, 2024). The ESHRE Good Practice Recommendations on IVF add-ons have emphasized that many adjunctive technologies, such as time-lapse imaging, the technology underpinning many AI-assisted embryo assessment tools, lack high-quality evidence of clinical benefit and are often marketed in ways that may mislead patients (ESHRE Add-Ons Working Group *et al.*, 2023). The Human Fertilisation and Embryology Authority (HFEA) has also rated time-lapse incubator with automated analyses of embryos as ‘black’ for improving the chances of pregnancy, stating that no established evidence show this add-on has effect on treatment outcome (ESHRE Add-Ons Working Group *et al.*, 2023; HFEA, 2023). Given that IVF success rates are a critical factor in decision-making for infertility patients (von Estorff *et al.*, 2024) such false marketing is particularly concerning.

False marketing of AI-assisted IVF services may significantly impact patients’ interest in IVF treatment, because patients often endure significant physical, psychological, and social pressures, making them especially vulnerable to deceptive or overly optimistic claims (The Lancet, 2024). In societies where traditional family structures prevail, infertility can also carry social stigma, increasing the risk of psychological distress, including depression (Taebi *et al.*, 2021). When providers emphasize favorable IVF prognoses aided by AI in their marketing, patients may be swayed toward inadequately validated solutions, potentially leading to disappointment, financial loss, and emotional harm.

From a legal perspective, legislation on medical devices, as well as laws on the promotion of healthcare services and consumer marketing in all countries where the reviewed AI-assisted IVF companies are registered, generally includes rules prohibiting misleading or false advertising of healthcare services and medical devices. For example, according to Article 7 of the EU Medical Device Regulation (MDR), advertising of medical devices should align with the intended purposes of the devices and should not mislead consumers. Therefore, unsubstantiated claims about AI-assisted IVF products or services as superior to conventional approaches and leading to higher pregnancy rates could be viewed as false advertising.

Generally, we recommend that AI providers avoid unproven claims that the deployment and use of AI are associated with improved clinical outcomes or increased fertility rates to ensure compliance with truthful advertising regulations. They should further avoid ambiguous statements and inaccuracies in their marketing of AI-assisted IVF services that may lead patients to take unnecessary risks or impair their ability to make informed health care decisions (Shaheen, 2025). To enforce these standards, regulatory bodies need to conduct a strict review of marketing practices for AI tools used in IVF, based on the latest evidence regarding the clinical effectiveness of these products.

### Potential risks are downplayed

Our study found that risks associated with the use of AI in IVF were significantly downplayed as service provider websites mentioned no risks at all. Yet, the scientific literature has shown that AI use in this context carries certain risks for patients. For example, AI systems may exhibit biases, and expectations of high performance may not generalize across all patient populations (Kragh and Karstoft, 2021). Existing studies indicate that AI models for IVF suffer from limitations in training and validation due to reliance on closed, non-public datasets and lack of external validation (Salih *et al.*, 2023; AlSaad *et al.*, 2024). AI models trained on public datasets also face problems concerning inadequate sample representation. One study of HFEA’s dataset highlighted underrepresentation of specific patient subgroups in ART cohort studies, particularly older patients (40–44 years) and ethnic minorities (Black and Asian populations) (Kondowe *et al.*, 2025). More critically, AI-enabled assessment tools may dismiss viable embryos as low-quality, leading to unnecessary discard, or misclassify poor-quality embryos as high-grade, resulting in implantation failures and miscarriages (Thirumalaraju *et al.*, 2026).

Therefore, making patients aware of potential AI bias and errors is critical. When IVF providers use AI as a selling point in their promotions, while failing to acknowledge health-related risks associated with its use, patients may develop unrealistic expectations that negatively impact their reproductive or health decisions (Iniesta, 2025). Because DTC service models often bypass professional advice and oversight, users without adequate medical knowledge or understanding of AI systems may struggle to interpret AI-based DTC test results on their own (Stanczyk *et al.*, 2022).

Furthermore, the understatement of privacy risks on the websites or in privacy policies could potentially make consumers less cautious about the protection of their privacy rights. In the EU, the USA, and China, there are compliance requirements currently in place for health data usage and transfer (Pernot-Leplay, 2020). However, our investigation has discovered that most companies failed to address privacy protection properly. In particular, no companies posted privacy policies related to the use of AI products on their websites, which presents challenges for DTC AI-assisted IVF product users who seek to evaluate potential privacy risks before using relevant AI products or services.

AI-assisted IVF service providers should provide balanced descriptions of the benefits and risks associated with AI applications. Serving as an entry point for information disclosure and marketing, AI provider websites must disclose risks associated with their services and products, ensuring that patients have the best available scientific information before making treatment decisions. This is a basic requirement for AI providers to fulfill their medical advertising compliance obligations. Privacy risks, as a significant risk in AI applications, should also be a priority disclosure item. Privacy policies should inform the public about the collection and processing of personal data and provide detailed information on how individuals can exercise their data rights, especially concerning sensitive personal information. More accurate and balanced information regarding AI applications can enhance users’ trust in AI products, foster best practices, and contribute to responsible innovation within the IVF domain.

### Advertising compliance, patient education, and ethics review

AI providers should adhere strictly to advertising compliance rules and rigorous disclosure standards, providing relevant risk disclosures and avoiding statements that could be deemed false advertising or misleading representation. Achieving this balance requires providers to present both benefits and risks associated with AI use. Furthermore, claims regarding accuracy or success rates should be substantiated with scientific evidence. Providers should also refrain from using superlatives, such as ‘guaranteed’, ‘best’, or ‘revolutionary’, that may create unrealistic expectations. Ultimately, compliance should not be treated as a mere checkbox but as an ongoing commitment to truthful, transparent communication that respects patients’ right to make informed decisions based on accurate medical information.

In addition, AI providers may leverage their websites as patient education portals to effectively educate prospective patients about AI. We are not arguing that websites should replace clinical consultation, where clinicians can address patient-specific questions on AI and tailor information to individual circumstances. Rather, we recommend that websites serve a complementary function in shaping patients’ initial perceptions before consultation, influencing their decision to seek care, and addressing informational gaps after clinical visits (Cromack *et al.*, 2025). In this regard, IVF providers’ websites should provide a concise and layperson-friendly explanation of AI-assisted IVF products, clearly articulating AI’s specific tasks and performance limitations in IVF clinical settings (Bramstedt, 2025). Furthermore, we suggest providers respond to public concerns regarding the use of AI in IVF by disclosing their data-protection measures of data processing and providing a separate AI-specific privacy policy detailing data governance for clinical applications. Additionally, providers need to clearly define the AI’s specific role in IVF clinical decision-making to help patients’ decision-making in AI-assisted IVF treatments.

Lastly, to ensure that patient autonomy is respected and that informed consent is truly meaningful, regulatory agencies should establish ethics review mechanisms. AI-assisted IVF products and services should be reviewed by a third-party ethics review board. This recommendation is supported by emerging regulatory developments on AI development nationally and internationally. In China, for example, *Measures for the Ethical Review and Service of Artificial Intelligence Technology (Trial)*, promulgated in March 2026, explicitly require enterprises engaged in AI research and application to establish an ethical review mechanism and to assess ethical risks based on application scenarios, with particular emphasis on data bias, algorithm transparency and explainability, and privacy protection. Similarly, in Korea, the 2025 *AI Basic Act* classifies AI systems used in medical devices as ‘High-Impact AI’, which triggers mandatory transparency obligations and provides a framework for voluntary safety verification and certification, reinforcing the expectation that AI providers bear upstream responsibility for ethical oversight (National Assembly of Korea, 2025). While the patients’ informed consent process is mainly conducted by physicians at the IVF clinic that deploys AI systems, the proposed third-party ethics committee can play a critical intermediary role in two ways. First, it can construct a patient-centric AI communication strategy that incorporates patients’ perspectives and specific concerns into informed consent materials provided to clinics. Second, it can verify the accuracy and balance of AI-related information and claims before dissemination to clinics and the public, thereby mitigating misleading influences due to commercial mandates.

### Limitations

This study has several limitations that should be acknowledged. First, it does not encompass all companies offering AI-assisted products or services in the IVF sector. Moreover, as AI technologies and relevant regulatory developments continue to evolve, the issues we have identified may not exhaustively capture all concerns associated with research on and use of AI-assisted IVF products. Nonetheless, the dataset we collected represents critical issues regarding AI integration within the current IVF services landscape. Second, while our study provides an overview of existing AI-assisted IVF products and services, including DTC services, we cannot fully determine the companies’ actual compliance with relevant data protection rules, including informed consent requirements, as our analysis was primarily based on information publicly available on companies’ websites. Website disclosures, however, remain a primary source of patient-facing information and thus warrant serious scrutiny. Third, our analysis does not capture the technical development, supply chain, or hosting arrangements of the AI tools. Whether AI systems are developed in-house or by external providers, and whether they are hosted on company or third-party servers, are important questions for future research and regulatory oversight. Finally, we acknowledge the dynamic nature of website content. Due to real-time updates, the specific product features, marketing materials, and technical disclosures observed during our investigation may not reflect the latest information on the websites under consideration. It is possible that some questionable marketing statements identified in this study have since been corrected. Despite these dynamic factors, the substantive issues regarding the portrayal of AI-assisted IVF services we have identified can inform both scholarly and policy discussions on ethics oversight and governance.

## Conclusions

Our study examined how AI-assisted IVF providers present their products and services on their websites and identified key ethical, legal, and public communication issues arising from these representations. We scrutinized how inaccurate representations of benefits and risks associated with the integration of AI applications in IVF might impact patients’ reproductive health decision-making and their rights to informed consent and privacy. Our study indicates that disclosure about how AI tools are used in treatment is insufficient, and patient-facing explanations on the websites lack important specifics, which could negatively affect patient autonomy in decision-making. Moreover, we highlight risks posed by misleading claims and overstated effectiveness of technological innovation that can diminish patients’ awareness of health and privacy risks and indirectly compromise decision-making about the appropriateness of certain procedures. Furthermore, for DTC AI-assisted IVF products, without guidance from fertility specialists, consumers using these products risk making uninformed health decisions, a problem exacerbated by the providers’ tendency to downplay risks.

When patients seek advanced reproductive treatments, accessible information about IVF and AI tools is equally important. Consequently, we advocate for the adoption of a responsible AI communication framework comprised of three components. First, AI providers must adhere strictly to truthful advertising regulations by balancing risk disclosure and avoiding false or misleading claims. Second, AI providers should proactively disclose AI system information through public-facing labels and layperson-friendly explanations on their websites, including separate privacy policies for data governance. Third, to ensure patient autonomy and meaningful informed consent, AI providers themselves should establish ethics review mechanisms to verify the accuracy of their claims and develop patient-centric communication materials for clinical use. Establishing this communication framework is necessary to cultivate public trust and AI literacy, which are essential for responsible development of AI-assisted IVF technology and services.

## Data Availability

The dataset used and analyzed for this study is publicly available via Figshare at: https://doi.org/10.6084/m9.figshare.33472525.
